# High expression of L-type amino acid transporter 1 as a prognostic marker in bile duct adenocarcinomas

**DOI:** 10.1002/cam4.272

**Published:** 2014-06-02

**Authors:** Nobuyuki Yanagisawa, Kiyomi Hana, Norihiro Nakada, Masaaki Ichinoe, Wasaburo Koizumi, Hitoshi Endou, Isao Okayasu, Yoshiki Murakumo

**Affiliations:** 1 Department of Pathology, Kitasato University School of MedicineKanagawa, 252-0374, Japan; 2 Department of Pathology of Biological Responses, Kitasato University Graduate School of Medical ScienceKanagawa, 252-0374, Japan; 3 J-Pharma Co. Ltd.Kanagawa, 230-0046, Japan; 4 Department of Gastroenterology, Kitasato University School of MedicineKanagawa, 252-0374, Japan; 5 Department of Pharmacology and Toxicology, Kyorin University School of MedicineTokyo, 181-8611, Japan

**Keywords:** Bile duct adenocarcinoma, CD98, immunohistochemistry, Ki-67, L-type amino acid transporter, prognosis

## Abstract

Oncocytic L-type amino acid transporter (LAT) 1 may be a prognostic indicator and target of new molecular therapeutic agents against malignancies. To investigate whether LAT1 expression influence the outcomes of patients with bile duct cancer, the expression of LAT1, LAT2, CD98, and Ki-67 was investigated immunohistochemically in 134 surgically resected bile duct adenocarcinomas, including 84 distal extrahepatic bile duct adenocarcinomas, 21 hilar cholangiocarcinomas, 15 intrahepatic cholangiocarcinomas, and 14 ampullary adenocarcinomas. LAT1 expression was weakly correlated with CD98 expression and Ki-67 labeling index (LI). Kaplan–Meier analysis showed a significant difference in prognosis between patients with bile duct adenocarcinomas having LAT1-high and -low scores, whereas LAT2 and CD98 expression and Ki-67 LI were not predictive of poor prognosis. Prognosis tended to be worse in patients having tumors with LAT1-high/LAT2-low than LAT1-low/LAT2-high scores (*P* = 0.0686). Multivariable analyses revealed that LAT1 expression, surgical margin, pT stage were independent prognostic factors. In conclusion, aberrant overexpression of LAT1 in bile duct adenocarcinoma predicts poor prognosis, suggesting that LAT1 may be a potential target of anticancer therapy.

## Introduction

Bile duct carcinoma (BDC) has one of the worst prognoses of human malignancies. Although obstructive jaundice may appear at an early stage, the prognosis of patients with advanced BDC remains generally poor, with 5-year survival rates of around 30–40% 1,2. The International Agency for Research on Cancer (IARC) has classified BDC into three types by anatomical location: intrahepatic cholangiocarcinoma (ICC), hilar cholangiocarcinomas (HCs), distal extrahepatic bile duct adenocarcinoma (EHC). While, ampullary adenocarcinoma (AC) has described as one of the tumors of the small intestine 3.

System L conveys the Na^+^-independent transport of large branched and aromatic neutral amino acids in almost all types of cells 4,5. L-type amino acid transporters (LATs) are responsible for the transport of large neutral amino acids such as leucine. Most of these transporters consist of two subunits, a light chain, including LAT1 (SLC7A5) and LAT2 (SLC7A8), and a heavy chain (CD98/4F2hc), located in the cell membrane 4,5. LAT2 is widely expressed in normal cells, including small intestine epithelial cells and proximal tubules of the kidney, suggesting it plays an important role in transepithelial amino acid transport 4,5. In contrast, LAT1 is expressed in many carcinoma cells, including prostatic, esophageal, gastric, pulmonary, and pancreatic carcinomas 6–10. Furthermore, some fetal cells express LAT1, suggesting that LAT1 has an aspect of oncofetal protein 11. We previously demonstrated that LAT1 expression could be a reliable prognostic marker in prostatic carcinoma and was correlated with the Gleason histological grading system 6. Further, we recently reported that high LAT1 expression was predictive of poorer prognosis in patients with pancreatic ductal adenocarcinomas, independent of cellular proliferation activity such as Ki-67 LI 10. These findings strongly suggested that LAT1 overexpression is associated with the aggressive phenotype of malignant tumors. We therefore assayed LAT1 expression in BDCs to determine whether altered LAT1 expression is related to the malignant behavior of BDCs.

## Material and Methods

### Patients and tissue samples

Tissue samples were obtained from 134 consecutive patients with BDC, including 84 EHCs, 21 HCs, 15 ICCs, and 14 ACs, who underwent surgical resection at Kitasato University East Hospital from 1993 to 2012. All tumors were classified by IARC/World Health Organization (WHO) histological typing as adenocarcinomas and by UICC pT classification for invasion 3,12. Of the patients with ICC, those with cholangiolocellular carcinoma that is a more primitive subtype resembling ductules or canals of Hering, and combined hepatocellular carcinoma were excluded. All of the resected specimens had been fixed in 10% buffered formalin, divided into 5 mm thick slices and embedded in paraffin. The largest tumor section from each patient was selected and used for hematoxylin-eosin staining and immunohistochemical analyses.

### Immunohistochemistry

Tissue sections 4 *μ*m thick were stained immunohistochemically as described 6. Briefly, endogenous peroxidase was blocked with 1% hydrogen peroxide in methanol for 30 min. After retrieving antigenic reactivity, the sections were incubated overnight at 4°C with primary antibodies, including mouse monoclonal anti-LAT1 (2 *μ*g/mL, J-Pharma Co., Ltd., Kanagawa, Japan), rabbit polyclonal anti-LAT2 (2 *μ*g/mL, Trans Genic Inc., Kumamoto, Japan), mouse monoclonal anti-CD98 (clone H-300, 1:200; Santa Cruz Biotechnology Inc., Dallas, TX), and mouse monoclonal anti-Ki-67 (1:100; Dako, Glostrup, Denmark). Anti-LAT1 and anti-LAT2 antibodies were confirmed the specificities previously 6,13. After incubation with peroxidase-labeled polymer (ChemMate EnVision kit, Dako) for 30 min, 3,3′-diaminobenzidine (DAB) was applied as the chromogen. Nuclei were counter-stained with 0.3% methyl green.

Selected samples were doubly immunostained with antibody to LAT1 or LAT2 and Ki-67 to compare their expression in the same cells. Following incubation with anti-LAT1 or anti-LAT2 antibody and DAB as above, antigen was retrieved by treatment in a microwave oven for 5 min. Following incubation with anti-Ki-67 antibody (1:50), the sections were reacted with a different chromogen, NiCl_2_-DAB.

Double immunofluorescence staining of some sections was performed to identify LAT1 and LAT2-positive cells. Deparaffinized histological sections were incubated with primary anti-LAT1 antibody overnight at 4°C and subsequently with fluorescein isothiocyanate-conjugated goat anti-mouse IgG (H+L) (1:1000, Molecular Probes, Invitrogen, Carlsbad, CA) at room temperature for 30 min. The sections were washed with phosphate-buffered saline (PBS), incubated with anti-LAT2 antibody overnight at 4°C and subsequently incubated with rhodamine-conjugated goat anti-rabbit IgG (1:200, Molecular Probes, Invitrogen) at room temperature for 30 min. For staining with antibody to LAT1, sections were microwave treated in 0.01 mol/L citrate buffer, pH 6.0, for 5 min prior to incubation with primary antibody. After washing with PBS, the slides were observed under a fluorescence microscope (Olympus BX61 + UCB + DP71, Tokyo, Japan).

### Evaluation of immunohistochemical staining

**Figure 1:**
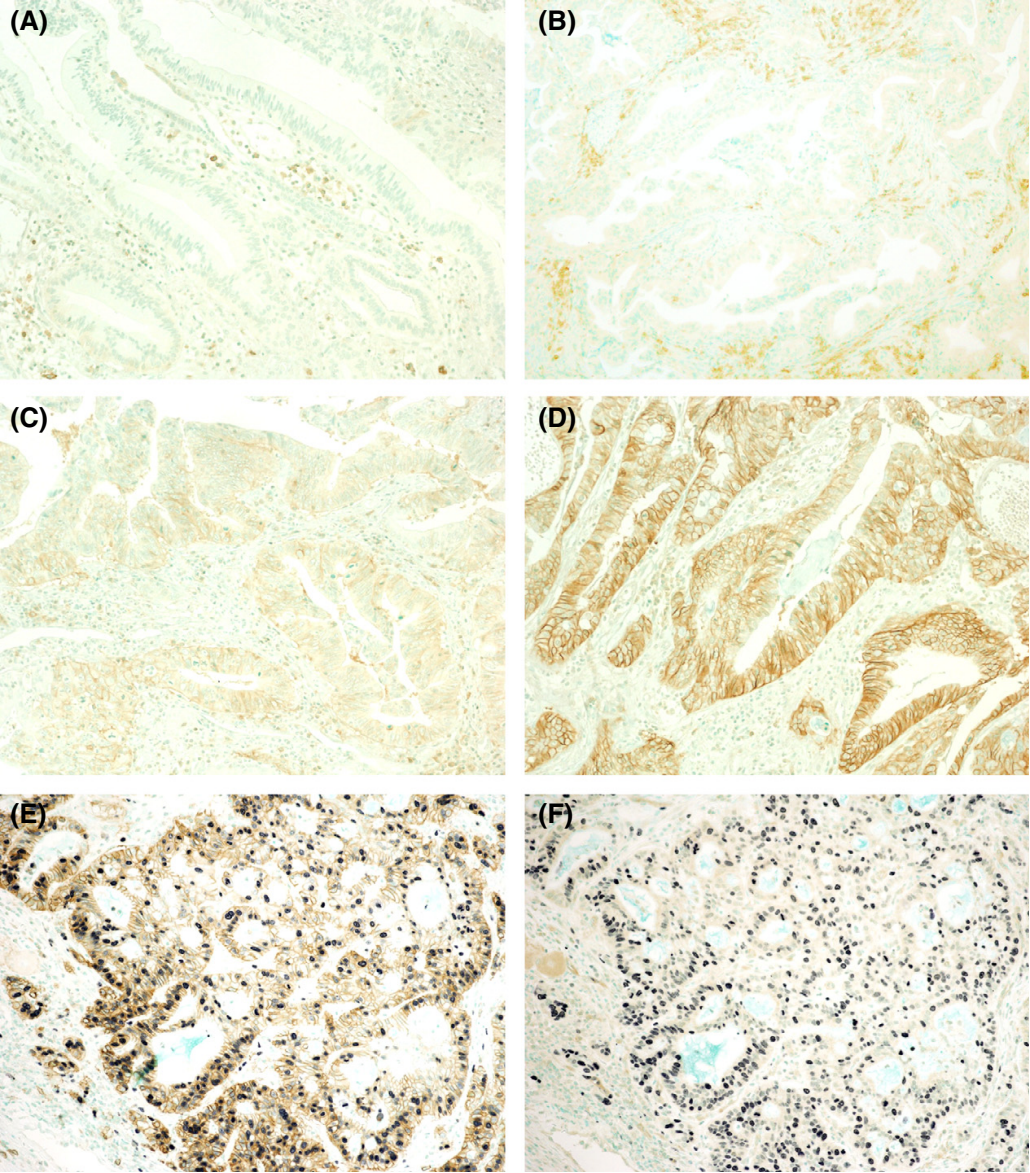
L-type amino acid transporter (LAT) 1 expression in bile duct carcinoma (BDC) cells analyzed by immunohistochemistry. Based on the immunointensity of the carcinoma cell membrane, four categories were defined: A, 0, no staining; B, 1, weak or patchily positive staining; C, 2, moderate cell membrane staining; and D, 3, intense complete membrane staining. Activated lymphocytes also showed LAT1 expression. E and F, double staining for LAT1 (E) or LAT2 (F) and Ki-67 in a representative patient with BDC. Many carcinoma cells that express Ki-67 (nuclear staining with NiCl_2_-DAB, blue) coexpress LAT1 (membranous staining with DAB, brown), but not LAT2. Slides were counter-stained with methyl green solution. Original magnification, ×100 (A through D), ×200 (E, F).

Expression of LAT1, LAT2, and CD98 was assessed as described 14, with minor modifications 6,10. Based on the immunointensity of the tumor cell membranes, four categories were defined: 0, no staining; 1, weakly or patchily positive; 2, moderate; and 3, intense complete membrane staining (Fig.1A–D). The percentage staining of the tumor area was classified as: 0, none; 1(focal), 1–10%; 2(partial), 11–30%; and 3(diffuse), >30%. Immuno-reactive scores were calculated by multiplying the scores for the area and highest intensity of positivity. All slides were scored by two pathologists (NY and IO) blinded to clinical information, with any disagreements resolved by further review and consensus. Immuno-reactive scores of 9 were classified as high and those of 0 to 6 as low. The number of Ki-67 positive cells was counted in at least 1000 cells of a mainly invasive area, with Ki-67 LI calculated as a percentage. Ki-67 LIs < 40% and ≥40% were classified as Ki-67-low and Ki-67-high groups, respectively, based on the average value of Ki-67 LI in BDCs (35.3 ± 16.9%) and previous results 10,15.

### Statistical analysis

Data were expressed as mean ± standard deviation. Groups were compared using Fisher's Protected Least Significant Difference test as a post hoc test. Differences between Kaplan–Meier survival curves were evaluated by the log-rank test. Correlations among LAT1, LAT2, and CD98 scores and Ki-67 LI were analyzed using Spearman's rank correlation coefficient test, and the relationships between the expression of these proteins and clinico-pathological factors were analyzed using Chi-square tests. Cox proportional hazard analysis was used to assess survival data. Factors significant on univariable analyses were examined by multivariable analysis. StatView software (version 5.0; Abacus Concepts Inc., Berkeley, CA) was used for all statistical analyses, with *P*-values <0.05 considered statistically significant.

### Ethics approval

Tissue samples were used with written informed consent of the patients. The study was approved by the Kitasato University School of Medicine and Kitasato University Hospital Ethics Committee (B05-34).

## Results

### Patient characteristics

Patient characteristics are summarized in Table 1. The 134 BDC patients included 94 men and 40 women, of mean age 65.3 ± 8.9 years (range 38–83 years). Of the tumors, 10 (7%) were classified as pT1, 63 (47%) as pT2, 57 (43%) as pT3, and 4 (3%) as pT4. Sixty-eight (51%) were classified as well-, 44 (33%) as moderately, and 22 (16%) as poorly differentiated adenocarcinomas 3. Of the 134 patients, 69 (52%) died of the disease after surgery.

**Table 1:** Patient demographics

Characteristics	All patients (*n* = 134)	AC (*n* = 14)	EHC (*n* = 84)	HC (*n* = 21)	ICC(*n* = 15)
Age	65.3 ± 8.9	66.0 ± 11.5	65.8 ± 8.4	63.5 ± 7.8	64.4 ± 10.7
Male:Female	94:40	11:3	60:24	13:8	10:5
Diff. (W/M/P)	68/44/22	11/3/0	38/27/19	15/4/2	4/10/1
pT stage					
T1	10	3	5	2	0
T2	63	6	27	18	12
T3	57	5	48	1	3
T4	4	0	4	0	0
LN metastases	62	4	46	7	5
R0 resection	79	13	51	7	8

AC, ampullary adenocarcinoma; Diff., differentiation; EHC, distal extrahepatic bile duct adenocarcinoma; HC, hilar cholangiocarcinoma; ICC, intrahepatic cholangiocarcinoma; LN, lymph node; M, moderately; P, poorly; W, well.

### LAT1 expression

**Figure 2:**
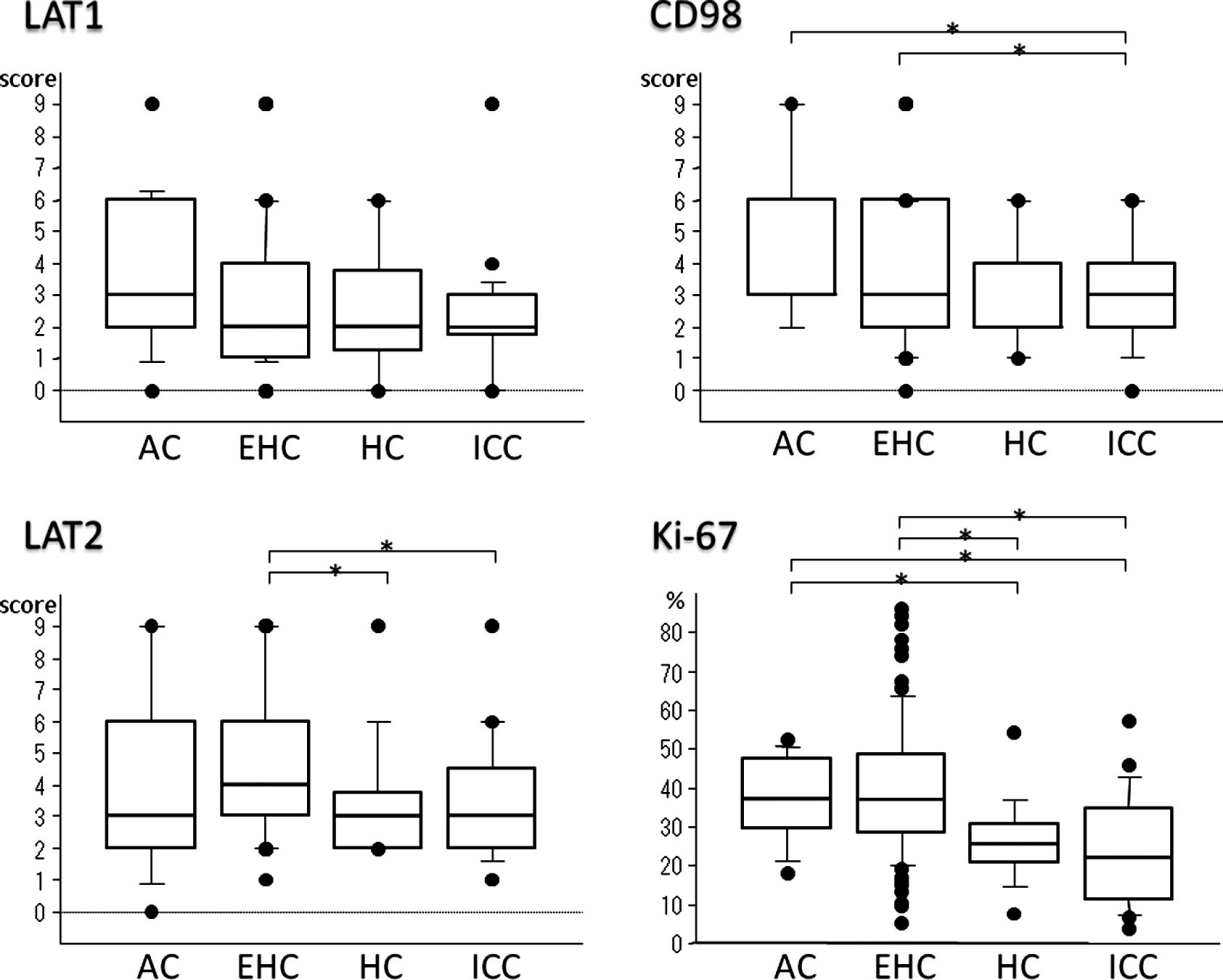
Comparison of LAT1, LAT2, and CD98 scores and Ki-67 LI of BDCs. LAT1 did not show significant differences in scores among ampullary adenocarcinomas (ACs), distal extrahepatic bile duct adenocarcinomas (EHCs), hilar cholangiocarcinomas (HCs) and intrahepatic cholangiocarcinomas (ICCs). EHCs showed significantly higher LAT2 and CD98 scores and Ki-67 LI compared with ICCs. *P-value indicates a significant difference.

LAT1 expression in normal epithelia of the bile duct was none to mild, although endocrine cells in the islets of Langerhans showed moderate to strong LAT1 membranous expression, and some activated lymphocytes showed moderate LAT1 expression. These cells were used as an internal control. Most BDCs showed aberrantly increased LAT1 expression, with an average LAT1 score of 3.0 ± 2.3, but no difference in LAT1 expression between invasive and noninvasive areas. LAT1 expression did not correlate significantly with the degree of tumor differentiation or tumor location, with scores in ACs, EHCs, HCs, and ICCs of 3.5 ± 2.4, 3.1 ± 2.4, 2.3 ± 1.9, and 2.6 ± 1.8, respectively (Fig.2).

### LAT2 and CD98 expression

Normal epithelia of the bile duct showed no to moderate LAT2 membranous expression without any polarity. Similar to LAT1, islet of Langerhans cells showed moderate to strong LAT2 membranous expressions, as did some lymphocytes, smooth muscle cells, and nerve sheath cells. The overall mean LAT2 score of BDCs was 4.5 ± 2.5, with scores in ACs, EHCs, HCs, and ICCs of 3.9 ± 3.1, 4.9 ± 2.4, 3.6 ± 2.0, and 3.5 ± 2.0, respectively (Fig.2).

CD98 expression showed the same pattern as LAT1 expression in normal cells and BDCs. The overall mean CD98 score of BDCs was 3.7 ± 2.1, with scores in ACs, EHCs, HCs, and ICCs of 4.3 ± 2.5, 3.9 ± 2.2, 2.9 ± 1.7, and 3.1 ± 1.8, respectively (Fig.2).

### Correlations among LAT1, LAT2, and CD98 expression, Ki-67 LI and clinico-pathological factors

**Figure 3:**
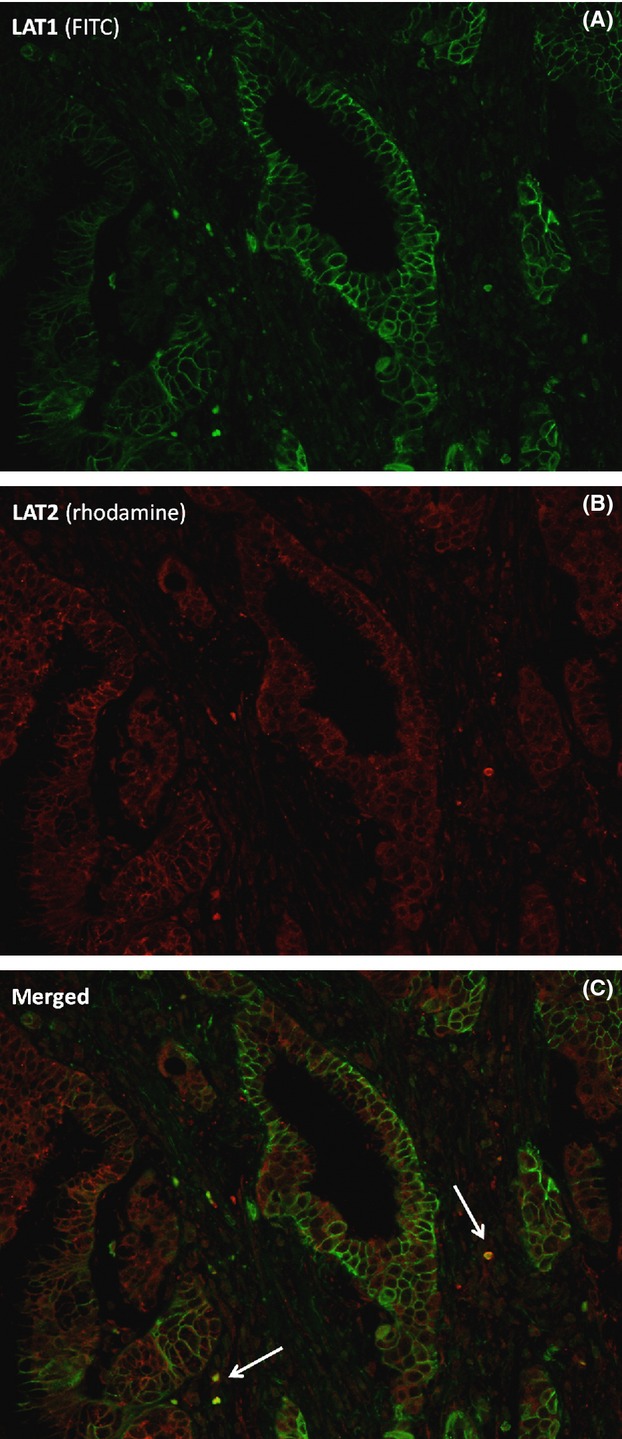
Double immunofluorescence staining with LAT1 and LAT2. Bile duct carcinoma (BDC) cells did not coexpress LAT1 (fluorescein isothiocyanate, green) and LAT2 (rhodamine, red), although some activated lymphocytes (arrow) showed both LAT1 and LAT2 expression (merged, yellow). Original magnification, ×200.

The correlations among LAT1, LAT2, and CD98 expressions and Ki-67 LI in BDCs are shown in Table 2. LAT1 and CD98 were positively correlated (*ρ* = 0.633, *P* < 0.0001), whereas correlations between LAT1 and Ki-67 LI (*ρ* = 0.314, *P* = 0.0003) and between Ki-67 LI and CD98 (*ρ* = 0.301, *P* = 0.0005) were weaker. No other correlations were found. Double staining showed that many cancer cells tended to coexpress LAT1 and Ki-67 (Fig.1E), but not LAT2 and Ki-67 (Fig.1F). Moreover, double immunofluorescence staining showed that BDC cells did not coexpress LAT1 and LAT2 (Fig.3). Lymph node metastasis was significantly correlated with LAT1 expression (*P* = 0.0159, data not shown), whereas none of the other clinico-pathological factors, such as lymphatic and vascular invasion and surgical margin status, did not correlate significantly with the level of expression of these proteins (data not shown). LAT1 (3.5 ± 2.7 vs. 2.5 ± 1.8, *P* = 0.0164) and CD98 (4.1 ± 2.2 vs. 3.3 ± 2.0, *P* = 0.0475) expression and Ki-67 LI (38.7 ± 19.3 vs. 32.3 ± 14.1, *P* = 0.0299) were significantly higher in the 62 BDCs with than the 72 without lymph node metastasis.

**Table 2:** Correlation coefficient for LAT1, LAT2, CD98 protein expressions and Ki-67 labeling index

	*ρ*	*P*
LAT1/LAT2	0.257	0.0030*
LAT1/Ki-67	0.314	0.0003*
LAT1/CD98	0.633	<0.0001*
LAT2/Ki-67	0.214	0.0136*
LAT2/CD98	0.260	0.0027*
Ki-67/CD98	0.301	0.0005*

LAT1, L-type amino acid transporter 1; LAT2, L-type amino acid transporter 2.

* *p*-value indicates a significant difference.

### Relationship with postoperative cause-specific survival

**Figure 4:**
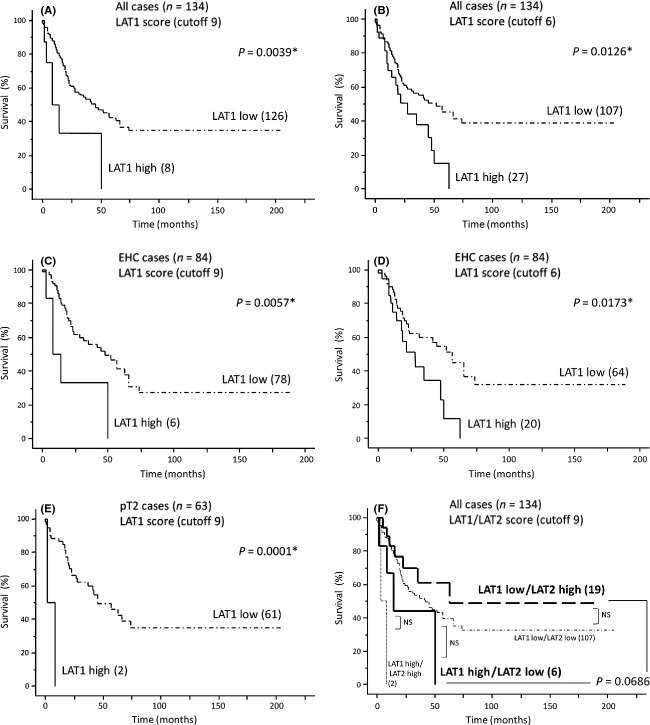
Cause-specific postoperative survival of patients with bile duct carcinoma (BDC) categorized by LAT1 expression. (A and B) Overall survival curves of patients with BDC divided by LAT1 scores with cutoffs of (A) 9 and (B) 6. (C–E) Overall survival curves of patients with distal extrahepatic bile duct adenocarcinoma (EHC) with LAT1 cutoffs of (C) 9 and (D) 6 and (E) of patients with pT2 stage tumors and an LAT1 cutoff of 9. In all log-rank tests, outcomes were significantly poorer in LAT1-high than LAT1-low groups. (F), Overall survival curves of BDC patients divided by LAT1 and LAT2 scores (cutoff 9). Prognosis tended to be poorer in LAT1-dominant (LAT1-high/LAT2-low; *n* = 6) than LAT2-dominant (LAT1-low/LAT2-high; *n* = 19) patients (*P* = 0.0686). *P-value indicates a significant difference. NS, not significant.

The 134 patients were followed up for a mean 38.9 ± 43.3 months after surgery (range 0–206 months). Patients with AC tended to have a better prognosis than patients with the other types of BDCs (data not shown). Based on LAT1 scores, BDCs were divided into two groups, 126 with LAT1-low (score 0–6) and 8 with LAT1-high (score 9) expression, with Kaplan–Meier analysis showing a significant difference in survival (*P* = 0.0039, Fig.4A). Using an LAT1 cutoff score of 6 divided these 134 patients into 27 with LAT1-high (score 6–9) and 107 with LAT-low expression, with the former having significantly poorer prognosis (*P* = 0.0126, Fig.4B). Similar results were observed for the 84 patients with EHC, in that the LAT1-high group, whether using a cutoff of 9 (*P* = 0.0057, Fig.4C) or 6 (*P* = 0.0173, Fig.4D) had a significantly poorer prognosis than the corresponding LAT1-low group. Among the 63 pT2 stage patients, those in the LAT1-high group with a cutoff of 9 (*P* = 0.0001, Fig.4E), but not a cutoff of 6 (*P* = 0.4554, data not shown), had a significantly poorer prognosis than those in the corresponding LAT1-low group. In contrast, the groups with high LAT2 (*n* = 21, *P* = 0.5908) and CD98 (*n* = 8, *P* = 0.1533) expression and high Ki-67-LI (≥40%, *n* = 23, *P* = 0.2706) showed no significant differences in survival outcomes, compared with their corresponding low groups (data not shown). Finally, prognosis tended to be poorer in patients with BDCs showing LAT1-high/LAT2-low than LAT1-low/LAT2-high expression (*P* = 0.0686, Fig.4F).

Univariate analysis showed that LAT1 expression, surgical margin status (hepatic, distal or peri-bile ductal margin), lymph node metastasis, and pT stage were prognostic in all patients with BDC (Table 3). Subsequent multivariable analysis showed that LAT1 expression, surgical margin status, and pT stage were independently prognostic of survival in these patients (Table 3). Univariate analysis of patients with EHC alone showed that LAT1 and CD98 expression, tumor differentiation, and lymph node metastasis were prognostic of survival (Table 4).

**Table 3:** Cox hazard analyses of cause-specific survival in bile duct adenocarcinomas (*n* = 134)

	Univariate		Multivariate	
		
Variable	OR (95% CI)	*P*	OR (95% CI)	*P*
Gender				
Male versus female (94:40)	0.980 (0.591–1.628)	0.9389		
Differentiation				
Well-mod. versus por. (112:22)	1.639 (0.911–2.951)	0.0994		
LAT1 score				
0–6 versus 9 (126:8)	3.246 (1.384–7.611)	0.0068*	2.670 (1.067–6.677)	0.0357*
0–4 versus 6–9 (107:27)	1.974 (1.141–3.415)	0.0150*		
LAT2 score				
0–6 versus 9 (113:21)	0.827 (0.410–1.666)	0.5942		
Ki-67 LI				
Cutoff 40% (91:43)	1.318 (0.802–2.164)	0.2758		
CD98 score				
0–6 versus 9 (97:37)	1.918 (0.767–4.796)	0.1639		
Surgical margin				
Negative/positive (79:55)	0.406 (0.251–0.657)	0.0002*	0.381 (0.233–0.621)	0.0001*
LN metastasis				
Negative/positive (72:62)	0.559 (0.347–0.900)	0.0168*	0.632 (0.384–1.038)	0.0698
pT stage				
pT1-2 versus pT3-4 (73:61)	0.560 (0.348–0.901)	0.0168*	0.604 (0.371–0.985)	0.0434*

CI, confidence interval; LAT1, L-type amino acid transporter 1; LAT2, L-type amino acid transporter 2; LI, labeling index; LN, lymph node; mod., moderately; OR, odds ratio; por., poorly.

* *P*-value indicates a significant difference.

**Table 4:** Cox hazard analyses of cause-specific survival in distal extrahepatic cholangiocellular carcinomas (*n* = 84)

Variable	Univariate		Multivariate	
	
	OR (95% CI)	*P*	OR (95% CI)	*P*
Gender				
Male versus female (60:24)	1.182 (0.627–2.231)	0.6052		
Differentiation				
Well versus mod.-por. (38:46)	1.979 (1.062–3.686)	0.0315*	1.615 (0.812–3.214)	0.1717
LAT1 score				
0–6 versus 9 (78:6)	3.492 (1.348–9.046)	0.0100*	1.457 (0.454–4.679)	0.5270
0–4 versus 6–9 (64:20)	2.168 (1.123–4.185)	0.0211*		
LAT2 score				
0–6 versus 9 (68:16)	0.921 (0.428–1.985)	0.8344		
Ki-67 LI				
Cutoff 40% (51:33)	1.737 (0.957–3.151)	0.0693		
CD98 score				
0–6 versus 9 (78:6)	4.811 (1.781–13.001)	0.0019*	3.538 (1.094–11.442)	0.0349*
Surgical margin				
Negative/positive (51:33)	0.607 (0.333–1.106)	0.1028		
LN metastasis				
Negative/positive (38:46)	0.525 (0.286–0.964)	0.0376*	0.707 (0.356–1.404)	0.3217
pT stage				
pT1-2 versus pT3–4 (32:52)	0.643 (0.340–1.214)	0.1733		

CI, confidence interval; LAT1, L-type amino acid transporter 1; LAT2, L-type amino acid transporter 2; LI, labeling index; LN, lymph node; mod., moderately; OR, odds ratio; por., poorly.

* *P*-value indicates a significant difference.

## Comment

LAT1 has been reported to be expressed in cell membranes of cancer cells of many organs 4,6–10,16, being thought to actively take up some essential amino acids. In contrast, many normal cells ubiquitously express LAT2, the second system L isoform 5,17. In contrast to LAT2, LAT1 transports both large and small neutral amino acids. The specificity of our monoclonal antibody against LAT1 was confirmed with an adsorption test using synthetic peptides and western blotting 6. Using this monoclonal antibody, we found that high-LAT1 expression was associated with poor prognosis in patients with BDC, similar to findings in other cancers 6–10. Several LAT1 inhibitors have been found to inhibit the growth of cancer cell lines. For example, one of these inhibitors, JPH203 (KYT-0353), significantly inhibited the growth of human colon cancer cells in vitro and in vivo 18, and 2-aminobicyclo-(2,2,1)-heptane-2-carboxylic acid, a second inhibitor, reduced the viability of lung cancer cells 19, suggesting that LAT1 inhibitors may be clinically useful as cancer chemotherapy. JPH203 is acetylated by hepatic cells, and this metabolic product, *N*-acetyl-JPH203, which also inhibits LAT1 20, is excreted and accumulates in the bile 21. These results strongly suggest that JPH203 is effective especially against BDCs that express high levels of LAT1.

Factors previously shown prognostic of survival in patients with BDC include lymph node metastasis and surgical margin status 22–25. Our results indicate that LAT1 overexpression may also be prognostic of poor survival in patients with BDC, especially in pT2 BDC or EHC. High LAT1 expression was associated with poorer prognosis in patients with negative (*P* = 0.0556) and positive (*P* = 0.0009) surgical margins (data not shown) and in patients with negative (*P* = 0.0004) and positive (*P* = 0.1546) for lymph node metastasis (data not shown), indicating that high LAT1 expression is an individual prognostic factor. In addition, the levels of expression of mRNAs encoding fms-related tyrosine kinase 1/vascular endothelial growth factor receptor 1 (*FLT1/VEGFR1*), heparanase (*HPSE*), and epidermal growth factor receptor (*EGFR*) have been significantly associated with overall survival in patients with cholangiocarcinomas 26, although we did not examine these molecular markers in patients with BDC.

Similar to findings in lung and gastric cancers, we observed a significant correlation between LAT1 expression and Ki-67 LI 8,9,27, suggesting that proliferating cells may require many amino acids. The double immunostaining for LAT1 and Ki-67 showed that the two were coexpressed in the same tumor cells, consistent with our statistical analysis. In addition, overexpression of LAT1 in glioma cells with low endogenous LAT1 expression was found to significantly enhance the rates of tumor cell growth 28. These results suggest a direct correlation between LAT1 expression and proliferative activity. However, the prognosis of BDC patients with high and low Ki-67 LI did not differ, for reasons not yet known. Nevertheless, our results indicate that LAT1 expression in BDC may be a new prognostic marker in these patients, independent of Ki-67 LI.

Although we and others have reported that LAT1 and LAT2 are expressed in some organs 11, double immunofluorescence staining demonstrated that few cells expressed both 11. We found that BDC did not coexpress LAT1 and LAT2, and there was no correlation between LAT1 and LAT2 scores. Interestingly, LAT1-high/LAT2-low expression tended to show poorer prognosis than LAT1-low/LAT2-high, although the difference was not significant. These results indicate that the oncocytic LAT1-dominant expression correlates with a more aggressive phenotype in malignancies. Because few normal cells express LAT1, LAT1 inhibitors may not affect normal organs, reducing side effects.

In conclusion, our findings suggested that elevated LAT1 expression in BDC is a novel biomarker for high-grade malignancy. Some LAT1 inhibitors have repressed cancer cell proliferation. Therefore, inhibition of LAT1 function may be a potential therapeutic strategy for BDC and other human cancers.
